# Long-term glycemic variability and the risk of cardiovascular diseases in type 2 diabetic patients: Effect of hypothetical interventions using parametric g-formula in a population-based historical cohort study

**DOI:** 10.1371/journal.pone.0319975

**Published:** 2025-05-28

**Authors:** Khadije Maajani, Ensieh Nasli-Esfahani, Noushin Fahimfar, Ali Sheidaei, Mohammad Ali Mansournia, Kamran Yazdani

**Affiliations:** 1 Department of Epidemiology and Biostatistics, School of Public Health, Tehran University of Medical Sciences, Tehran, Iran; 2 Diabetes Research Center, Endocrinology and Metabolism Clinical Sciences Institute, Tehran University of Medical Sciences, Tehran, Iran; 3 Osteoporosis Research Center, Endocrinology and Metabolism Clinical Sciences Institute, Tehran University of Medical Sciences, Tehran, Iran; Kerman University of Medical Sciences Physiology Research Center, IRAN, ISLAMIC REPUBLIC OF

## Abstract

**Background:**

Harmful effects of long-term HbA1c and fasting plasma glucose (FPG) variability on cardiovascular diseases (CVD) have not been causally examined. We employed a parametric g-formula to estimate the causal effect of HbA1C and fasting plasma glucose (FPG) variability on CVD.

**Methods:**

This retrospective cohort study was conducted on 2078 patients with type 2 diabetes who were free of CVD and aged >18 years at the entrance to the clinic (2017–2022), with at least three HbA1c and FPG measurements. Variability was calculated using standard deviation (SD), and coefficient of variation (CV). We used the parametric g-formula to estimate the 5-year risk, risk ratio, and risk difference of CVD under different deciles of HbA1c-SD/CV, FPG-SD/CV, HbA1C levels (≤5%, 5 to ≤7%, and >7), and joint exposure to different deciles of HbA1c-SD and HbA1c values, adjusted for time-varying confounders that are affected by prior exposure.

**Results:**

The observed and simulated 5-year risk of CVD under no intervention were 11.6% (95% CI: 10.3, 13.1) and 11.03% (95% CI: 10.2, 12.6) for HbA1C-SD model. The estimated 5-year risk of CVD was increased from the 8.01% (95% CI: 7.5, 10.1%) in the first decile to 15.2% (95% CI: 14.1, 17.7%) in the tenth decile of HbA1c-SD. The results for FPG-SD were similar. Within the stable level of HbA1c (5 to ≤7%) the risk ratio increased from 1.37 (95% CI: 1.19, 1.48) in the first decile to 2.76 (95% CI: 2.06, 3.09) in the tenth decile of HbA1c-SD. Under a joint intervention of HbA1c <5% and the first decile of HbA1c-SD, CVD risk decreased by 6.4% (95%CI: 4.9, 7.3%) compared to the natural course.

**Conclusions:**

Even within a stable HbA1c level, long-term glycemic variability may be a strong predictor of CVD.

## 1. Introduction

In patients with type 2 diabetes (T2D), glycemic variability (GV), a form of dysglycemia [1], is now thought to be an independent predictor of micro- and macrovascular complications, including cardiovascular diseases (CVD) [2]. emerging evidence suggests that GV could be used along with HbA1c, which is the gold standard, to monitor glycemic status [3–6]. Short-term GV is defined by measuring the fluctuations of fasting plasma glucose (FPG) and postprandial glucose (PPG) over hours and days; moreover, long-term GV is defined based on the changes of the amounts of serial FPG, PPG, and HbA1c, over months or years [4,5].

The effect of time-varying exposures and confounders has frequently been estimated in the aforementioned studies using conventional regression models, such as time-dependent Cox regression and generalized estimation equations [7–9]. However, these approaches may result in biased estimates because they are unable to appropriately adjust for time-varying confounders influenced by prior exposure [10]. To overcome these problems, causal methods, such as the parametric g-formula, have been proposed. This method offers the additional advantage of simultaneously examining hypothetical interventions on multiple risk factors [11]. Hence, we aimed to estimate the causal effect of long-term FPG and HbA1c variability on the risk of CVD using this method.

## 2. Methods

### 2.1. Study design and participants

Our study had a retrospective cohort design, and we used data from the electronic health records (EHR) of diabetes clinic No.1 of Tehran University of Medical Sciences in Iran. The cohort included all diabetes patients, with follow-up visits scheduled every three-months. All patients with type 2 diabetes (T2D) who were enrolled between March 21, 2017, and March 20, 2022, aged 18 years or older, with no history of cardiovascular diseases based on their self-reported baseline history, and with at least three HbA1c and FPG measurements, met our eligibility criteria. patients who experienced CVD events between the entrance to the clinic (baseline) and the first visit were excluded, as at least two measures of HbA1c and FPG were needed to compute GV. any occurrence of CVD after the second visit was considered our outcome of interest. this paper was written in accordance with strengthening the reporting of observational studies in epidemiology (STROBE) checklist for cohort studies [12] and the checklist for accurate reporting in medical research statistics [13,14].

This study, which was part of a PhD dissertation in epidemiology at Tehran University of Medical Sciences was approved by the Ethics Committee of the School of Public Health (ID: IR.TUMS.SPH.REC.1401.129). We had official permission from the Diabetes Research Center of Tehran University of Medical Sciences to use the data for this study. Data for this research were accessed on November 4, 2022 and the dataset contained no identifying information about the participants.

### 2.2. Measurements

#### 2.2.1. Specifying time intervals.

To obtain a consistent time interval in our data extraction, we rounded intervals shorter or longer than three-month as follows: a) time intervals of two to four months from the prior visit were considered three months, b) intervals between five and seven months were considered six months, c) if there was one missing visit between three-month intervals, it was filled using the last observation carried forward (LOCF) method, and d) if the time interval between the imputed visit (using LOCF) and the next visit was more than seven months, the remaining visits were considered missing. This method was also used to fill in other missing values.

#### 2.2.2. Glycemic variability (exposures).

To estimate the long-term glucose variability at each visit we calculated the standard deviation (HbA1c-SD, FPG-SD), and the coefficient of variation (HbA1c-CV, FPG-CV) based on the values from the current and previous visits. Additionally, we used total 5-year values of HbA1c and FPG to estimate 5-year glucose variability.

#### 2.2.3. CVD (outcome).

The cardiovascular diseases (CVD) incident cases that recorded by physicians based on patient self-report, included non-fatal stroke and non-fatal coronary artery disease (CAD), which were our outcome of interest. We defined CAD as the presence of any of the following: coronary insufficiency, acute coronary syndrome, myocardial infarction, and angina pectoris. Furthermore, we considered the presence of any of following procedures: coronary artery bypass graft surgery, angioplasty, or percutaneous coronary intervention. Details of the outcome definition have been reported previously [15].

#### 2.2.4. Other variables (confounders).

The variables we extracted from EHR were as follows: age, sex, the duration of diabetes from diagnosis until enrollment, body mass index (BMI), and systolic and diastolic blood pressures (SBP, DBP), HbA1c (measured using High-performance liquid chromatography), FPG (measured using enzymatic calorimetric methods using glucose oxidase test), total cholesterol (TC), high-density lipoprotein (HDL), low-density lipoprotein (LDL), and triglyceride (TG) (enzymatic methods were used to determine the lipid profile). Additionally, data of prescribed medications were obtained at each visit, including oral glucose-lowering drugs (1. SGLT2-inhibitors, 2. Other oral glucose-lowering drugs), and injectable anti-diabetic medications (3. GLP1 agonists, 4. Insulin) as well as, 5. anti-hypertensive, 6. Lipid lowering and 7. Anti-platelet drugs. All used variables were measured at every visit, except for age, sex, duration of diabetes and which was recorded at baseline. Furthermore, lipid profile was measured at least twice a year.

All clinical and laboratory measurements were performed by the trained staff, details have been reported elsewhere [15].

### 2.3 . Statistical analysis

#### 2.3.1. Descriptive statistics.

The baseline characteristics of study participants were summarized as mean (SD) for quantitative variables and frequency (percent) for categorical variables. Because visit-to-visit GV is a time-varying variable, and describing the baseline characteristics across 20 visits would be confusing, we compared the baseline values of covariates in our study across the 10 deciles of 5-year HbA1c-SD, FPG-SD, Hb1Ac-CV, and FPG-CV using one-way ANOVA or non-parametric Kruskal-Wallis test, as well as chi-square test. however, we have only reported the results of the HbA1c-SD deciles.

#### 2.3.2. parametric g-formula.

**Fig 1:**
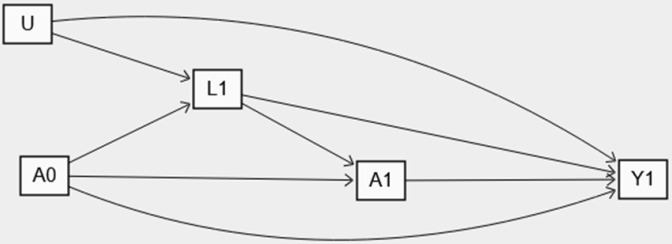
Causal directed acyclic graph (DAG) of the relationship between time-varying exposures for example, HbA1C-SD **(A),** **on the risk of cardiovascular diseases** **(Y)**. L stand for time-varying confounders (such as glucose lowering medications) and U indicates unmeasured risk factors, with only two visits, depicted by subscripts 0 and 1.

One of the major methodological challenges was correctly adjusting for time-varying confounders that are affected by prior exposures. For example, Fig 1 illustrates the relationship between time-varying exposures A (e.g., HbA1c-SD) and the risk of outcome Y (CVD) in two visits, depicted by subscripts 0 and 1. At the second visit, glucose-lowering medication (L_1_) is a time-varying confounder that affects HbA1c-SD (A_1_), and the risk of CVD (Y_1_), but L_1_ is also influenced by the prior HbA1c-SD value (A_0_). In such cases, using standard regression models to adjust for L_1_, a mediator variable between A_0_ and Y_1_, may introduce over-adjustment bias. Furthermore, if an unmeasured risk factor (U) affects the glucose-lowering medication (L_1_), adjusting for L_1_ equivalent to conditioning on a collider (between past HbA1c-SD and the unmeasured risk factor; U), potentially resulting in selection bias [10,16]. To address the biases resulting from conventional regression models, we used the parametric g-formula. This method is a model-based standardization for time-varying covariates and provides an unbiased estimation of the risk of outcomes under the assumptions of consistency, exchangeability, and positivity and correct model specification [11].

##### 2.3.2.1. Hypothetical intervention:

We used the parametric g-formula to estimate the 5-year risk of CVD for each of the following intervention and their combinations as joint hypothetical interventions:

1) Exposure to the first through tenth decile of HbA1c-SD, FPG-SD, HbA1c-CV and FPG-CV separately.
2) Exposure to HbA1c values categorized as: ≤ 5%, 5 to <7%, and ≥7%.
3) Exposure to HbA1c ≤ 5% combined with the first through tenth decile of HbA1c-SD respectively.
4) Exposure to HbA1c 5–7%, combined with the first through tenth decile of HbA1c-SD respectively.
5) Exposure to HbA1c ≥ 7% combined with the first through tenth decile of HbA1c-SD respectively.

##### 2.3.2.2. Implementing parametric g-formula:

The steps for performing the parametric g-formula were previously described in detail [17], and we summarized them as follows;

First, for each visit (between 2017 and 2022), we fitted regression models in two steps for each time-varying covariates (step 1a: covariates model), and CVD (step 1b: outcome model) using pooled person-time data. To account for censoring due to loss to follow-up, we fitted a parametric model (e.g., logistic) for censoring. Afterward, we used the parametric g-formula with simulated censoring to estimate the parametric Kaplan-Meier risk among simulated individuals who remain uncensored at each time point [18]. Table 1 demonstrates that each time-varying covariate was used as a dependent variable in the covariate model. We included all time-fixed covariates, baseline values, and the initial lag of time-varying covariates as independent predictors in both the covariate models and the outcome model.

**Table 1:** Summary of covariates used to model^**^ incidence of cardiovascular diseases during 5 years of follow-up in the type 2 diabetes patients in diabetes clinic (2017-2022).

Variable names	Examination visit assessed	Types of models used as dependent variable	As independent (Function form when used as predictor)
Time-fixed covariates			
Age	Baseline	Not predicted	Continuous
Sex	Baseline	Not predicted	2 categories
Duration of disease	Baseline	Not predicted	Continuous
Baseline BMI	Baseline	Not predicted	Continuous
Baseline SBP	Baseline	Not predicted	Continuous
Baseline DBP	Baseline	Not predicted	Continuous
Baseline HbA1c	Baseline	Not predicted	Continuous
Baseline FBS	Baseline	Not predicted	Continuous
Baseline Cholesterol	Baseline	Not predicted	Continuous
Baseline HDL	Baseline	Not predicted	Continuous
Baseline LDL	Baseline	Not predicted	Continuous
Baseline Triglyceride	Baseline	Not predicted	Continuous
Baseline SGL2 oral medication	Baseline	Not predicted	Binary^b^
Baseline of other oral medication	Baseline	Not predicted	Binary
Baseline GLP1 injection	Baseline	Not predicted	Binary
Baseline insulin	Baseline	Not predicted	Binary
Baseline Anti-hypertensive medication	Baseline	Not predicted	Binary
Baseline Anti-lipid medication	Baseline	Not predicted	Binary
Baseline Anti-platelet medication	Baseline	Not predicted	Binary
Time-varying covariates			
BMI	All visits	Linear regression	Continuous
SBP	All visits	Linear regression	Continuous
DBP	All visits	Linear regression	Continuous
HbA1c	All visits	Linear regression	Continuous
Visit to visit HbA1c SD	All visits	Linear regression	Continuous
Visit to visit HbA1c CV	All visits	Linear regression	Continuous
FBS	All visits	Linear regression	Continuous
Cholesterol	At least 2 times	Linear regression	Continuous
HDL	At least 2 times	Linear regression	Continuous
LDL	At least 2 times	Linear regression	Continuous
Triglyceride	At least 2 times	Linear regression	Continuous
SGL2 oral medication	All visits	Logistic regression	Binary
Other oral medication	All visits	Logistic regression	Binary
GLP1 injection	All visits	Logistic regression	Binary
insulin	All visits	Logistic regression	Binary
Anti-hypertensive medication	All visits	Logistic regression	Binary
Anti-lipid medication	All visits	Logistic regression	Binary
Anti-platelet medication	All visits	Logistic regression	Binary

** All covariates model included all time-fixed, time-varying and the first lag of time-varying covariates in the table.

Second, we simulated a cohort of patients (a random sample, n=10000) under hypothetical intervention strategy in five steps: 1. Use the observed value of covariate at baseline, 2. Use the coefficients from the parametric regression models in step 1a to predict the value of covariates at the next visit, 3. Change the value of exposures in each visit to those determined by hypothetical interventions; 4. Estimate the probability of CVD risk at each visit, using the coefficient from the outcome model in step 1b, 5. repeat steps 1–4 for each visit and estimate the population CVD risk under hypothetical interventions as subject-specific risk.

Third, to estimate the 5-year risk of exposure to different hypothetical interventions, we repeated steps 1–4. Additionally, to estimate the 95% confidence interval (CI), we used a nonparametric bootstrap with 200 samples.

### 2.4. Sensitivity analyses

The validity of our models was evaluated via sensitivity analyses: (1) we tested the model fit by comparing observed with predicted risk under no intervention, (2) we assessed the influence of covariates order in a covariate and outcome models on the final result, and (3) in the primary analyses, we entered continuous variables linearly, and in the secondary analyses, we entered them as polynomials (quadratic and cubic).

We performed all statistical analyses using R software version 4.4.1, with the g-formula package in R environment [19], available online (https://github.com/CausalInference/gfoRmula).

## 3. Results

### 3.1. Baseline characteristics

**Fig 2:**
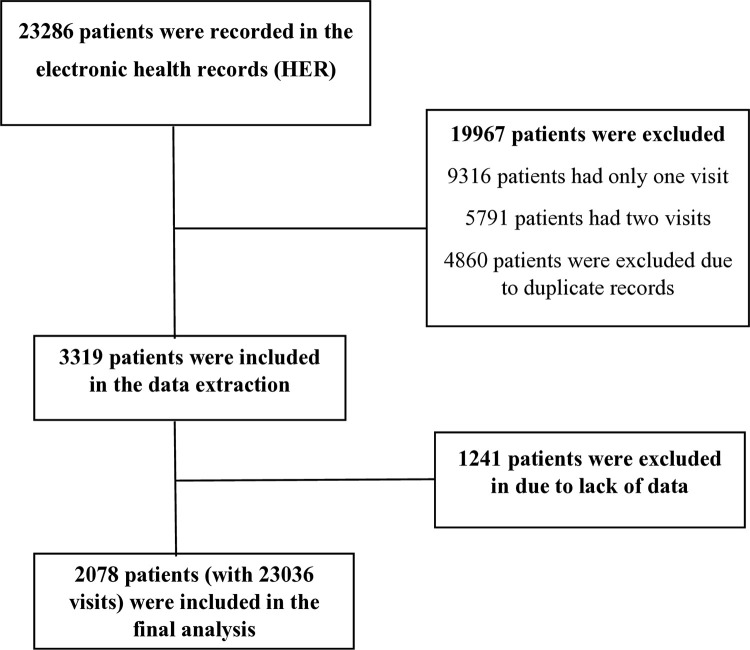
The flowchart of the inclusion of diabetic patients in the final analysis.

Among 3319 patients with T2D who had three or more visits, we included 2078 patients with 23036 observations in the analyses, Fig 2. Participants’ mean (SD) age was 64.7 (10.6) at baseline, and 55.2% were females.

We compared the baseline characteristics across different deciles of “5-year HbA1c-SD” presented in Table 2. The mean HbA1c-SD rose significantly from 0.23 ± 0.06 in the first decile to 2.1 ± 0.49 in the tenth decile. other GV index also rose significantly from first to the tenth decile. Moreover, the mean HbA1c, FPG, BMI, SBP, DPB, TC, LDL, and TG were significantly higher in the tenth decile than in the first decile, except for HDL which decreased from 43.9 ± 11.7 in the first decile to 39.3 ± 11.1 in the tenth decile. Similarly, the percentage of patients using glucose-lowering medicines, such as SGLT2 inhibitors, GLP1 agonists, and insulin, increased dramatically from the first to the tenth decile. The median follow-up duration was 32 (IQR: 28) months, with 280 CVD incidents occurring during the maximum 60-month follow-up period.

**Table 2:** Baseline characteristics according to the decile of 5-year HbA1c-SD in 2078 type2 diabetes patients in clinic of diabetes^*^.

Variables	HbA1c-SD decile										
	Decile 1 (n = 208)	Decile 2 (n= 208)	Decile 3 (n= 208)	Decile 4 (n= 208)	Decile 5 (n= 207)	Decile 6 (n= 208)	Decile 7 (n= 208)	Decile 8 (n= 208)	Decile 9 (n= 208)	Decile 10 (n= 207)	P-value
Age	67.1 ± 9.2	66.8 ± 10.2	64.9 ± 10.8	64.6 ± 9.8	64.3 ± 11.5	65.8 ± 10.1	66.1 ± 9.6	64.7 ± 10.5	63.02 ± 11.1	60.5 ± 11.7	<0.001^*^
Age category											<0.001^1^
< 65	82 (39.4)	83 (39.9)	105 (50.5)	100 (48.1)	103 (49.8)	83 (39.9)	94 (45.2)	98 (47.1)	107 (51.4)	134 (64.7)	
≥ 65	126 (60.6)	125 (60.1)	103 (49.5)	108 (51.9)	104 (50.2)	125 (60.1)	114 (54.8)	110 (52.9)	101 (48.6)	73 (35.3)	
Sex											0.09^1^
Male	92 (44.2)	93 (44.7)	87 (41.8)	90 (43.3)	99 (47.8)	89 (42.8)	101 (48.6)	86 (41.3)	82 (39.4)	113 (54.6)	
Female	116 (55.8)	115 (55.3)	121 (58.2)	118 (56.7)	108 (52.2)	119 (57.2)	107 (51.4)	122 (58.7)	126 (60.6)	94 (45.4)	
Duration of disease (year)	16.8 ± 8.6	17.3 ± 8.7	19.1 ± 9.4	18.5 ± 8.5	19.1 ± 8.1	19.6 ± 9.1	20.7 ± 8.7	20.3 ± 9.7	19.4 ± 9.4	15.4 ± 9.3	<0.001^*^
BMI	27.8 ± 4.8	27.2 ± 4.1	28.3 ± 4.02	28.5 ± 4.6	28.3 ± 5.01	28.5 ± 4.7	28.2 ± 4.6	29.03 ± 5.2	28.5 ± 5.3	28.4 ± 5.2	0.02^*^
SBP (mmHg)											
Median (IQR)	123.4 ± 15.9 125 (17)	126.1 ± 16 125 (20)	128.6 ±16.1 130 (20)	125.9 ± 19.3 127.5 (25)	126.6±17.5 125 (20)	130.1 ±17.4 130 (20)	128.4 ±17.6 126.5 (20)	128 ± 19.7 125 (25)	126.7 ±16.8 125 (21)	129.1 ±19.3 130 (20)	0.01^**^
DBP (mmHg)	76.01 ±9.03	75.8 ±9.04	76.6 ± 8.7	76.2 ± 9.8	75.7 ± 9	77.1 ± 9.6	75.6 ± 8.3	76.8 ± 10	76.5 ± 8.5	80.1 ± 9.5	<0.001^*^
HbA1c %	6.8 ± 0.82	7.01 ± 0.8	7.2 ± 0.9	7.5 ± 1.1	7.6 ± 1.03	7.7 ± 1.3	8.03 ± 1.3	8.4 ± 1.5	9 ± 1.7	10.6 ± 2.4	<0.001^*^
FPG, mg/dl	129.3 ±37.4	132.5±32.2	137.3±36.3	143 ±43.6	138.3 ±38	148.8 ±56.8	151.5 ±51.4	172.3 ±62.5	183 ±70.7	229.8±93.3	<0.001^*^
TC	149 ± 34.2	143.8 ± 31	145 ± 30.2	149.3 ±41.2	147.5 ±37.2	146.3 ±37	148.4 ±34.4	148.4 ±35.5	159.3 ±41.8	172.1 ±54.2	<0.001^*^
HDL	43.9 ± 11.7	44.2 ± 10.8	42.8 ± 11.3	41.8 ± 11.5	42.2 ± 11.1	42.6 ± 11.8	41.03 ± 9.3	41.3 ± 9.5	40.9 ± 10.2	39.3 ± 11.1	<0.001^*^
LDL	86 ± 34.4	78.8 ± 23.1	78.9 ± 23.3	85.4 ± 38.5	80.3 ± 26.6	80.26 ±28.5	80.8 ± 27.4	81.8 ± 27.6	91.3 ± 30.5	95.1 ±35.1	<0.001^*^
TG	139.7±79.9	136.8±61.8	147.4±83.4	149.2±71.2	146.3±79	148.5±71.5	152.2±74.6	158.7±91.7	160±101.1	194.5±122.6	<0.001^*^
Variables	HbA1c-SD decile										
	Decile 1 (n = 208)	Decile 2 (n= 208)	Decile 3 (n= 208)	Decile 4 (n= 208)	Decile 5 (n= 207)	Decile 6 (n= 208)	Decile 7 (n= 208)	Decile 8 (n= 208)	Decile 9 (n= 208)	Decile 10 (n= 207)	P-value
**Oral glucose lowering agent usage, yes (%)**											
SGLT2-inhibitors	1 (0.5)	5 (2.4)	1 (0.5)	2 (1)	2 (1)	3 (1.4)	3 (1.4)	4 (1.9)	3 (1.4)	8 (3.9)	0.18^1^
Another oral-glucose drug	185 (88.9)	187 (89.9)	182 (87.5)	182 (87.5)	157 (75.8)	177 (85.1)	174 (83.7)	174 (83.7)	173 (83.2)	167 (80.7)	0.003^1^
GLP1-agonist,	0	2 (1)	1 (0.5)	0	1 (0.5)	0	0	2 (1)	4 (1.9)	4 (1.9)	0.06^1^
Insulin usage,	38 (18.3)	33 (15.9)	51 (24.5)	50 (24)	73 (35.3)	68 (32.7)	89 (42.8)	85 (40.9)	94 (45.2)	92 (44.4)	<0.001^1^
**Other medication usage (%)**											
Blood pressure lowering	125 (60.1)	135 (64.9)	130 (62.5)	128 (61.5)	112 (54.1)	124 (59.6)	131 (63)	122 (58.7)	113 (54.3)	100 (48.3)	0.02^1^
Lipid-lowering	185 (88.9)	172 (82.7)	182 (87.5)	178 (85.6)	161 (77.8)	178 (85.6)	174 (83.7)	165 (79.3)	161 (77.4)	155 (74.9)	<0.001^1^
Platelet-lowering	156 (75)	162 (77.9)	145 (69.7)	156 (75)	146 (70.5)	149 (71.6)	157 (75.5)	143 (68.8)	131 (63)	115 (55.6)	<0.001^1^
**5-year glycemic variability**											
Hb1C-SD	0.23 ± 0.06	0.37 ± 0.03	0.47 ± 0.03	0.58 ± 0.03	0.68 ± 0.03	0.79 ± 0.3	0.90 ± 0.4	1.05 ± 0.05	1.32 ± 0.11	2.1 ± 0.49	<0.001^*^
HbA1c-CV	3.5 ± 0.9	5.4 ± 0.7	6.7 ± 0.8	7.9 ± 0.9	9.2 ± 1.1	10.3 ± 1.1	11.7 ± 1.5	13.1 ± 1.7	15.9 ± 2.3	23.7 ± 5.9	<0.001^*^
FPG-SD	15.3 ± 13.9 11.7 (9.03)	17.7 ± 10.3 15.1 (10.5)	22.8 ± 14.2 18.9 (13.8)	25.02 ± 13.8 22.6 (14.9)	30.5 ± 18.6 26.8 (16.2)	32.6 ± 16.2 29.3 (18.9)	33.9 ± 17.2 30.2 (19.8)	40 ± 19.2 36.8 (22.9)	46.6 ± 22.5 43.6 (25.5)	64.5 ± 30.05 63.3 (37.2)	<0.001^**^
FPG-CV	11.8 ± 11.6 9.7 (7.1)	13.7 ± 8.8 11.7 (7.8)	17.6 ± 15.3 14.3 (10.4)	18.6 ± 12.5 15.8 (10.6)	23.8 ± 20.4 19.2 (10.8)	23.5 ± 13.4 19.8 (12.9)	23.8 ± 13.4 20.4 (11.7)	24.6 ± 13 21.5 (13.8)	28.8 ± 19.9 23.2 (13.2)	29.7 ± 13.9 26.7 (12.9)	<0.001^**^

* Values are presented as mean ± standard deviation, median (Inter-quartile range), or as number (percent).

* One-way ANOVA,

** Kruskal-Wallis,

^1^Chi-square

Abbreviations: BMI, body mass index; SBP, systolic blood pressure; DBP, diastolic blood pressure; FPG, fasting plasma glucose; TC, total cholesterol; HDL, high-density lipoprotein; LDL; low density lipoprotein; TG, triglyceride

**Fig 3:**
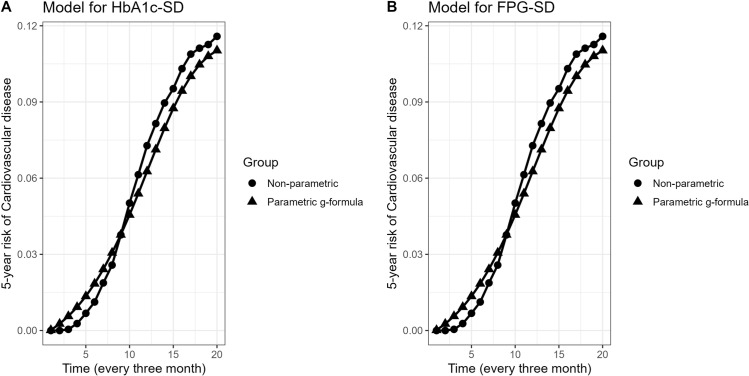
Comparing the 5-year risk of CVD under no intervention (observed risk) and simulated (g-form risk) for A) HbA1C-SD and B) FPG-SD model.

The observed risk in the data was 11.6% (95% CI: 10.3, 13.1), which was close to the simulated 5-year risk of CVD under no intervention for the HbA1c-SD model 11.03% (95% CI: 10.2, 12.6) and for the FPG-SD model 11.1% (95% CI: 10.3, 12.6), indicating satisfying model specification Fig 3.

**Fig 4:**
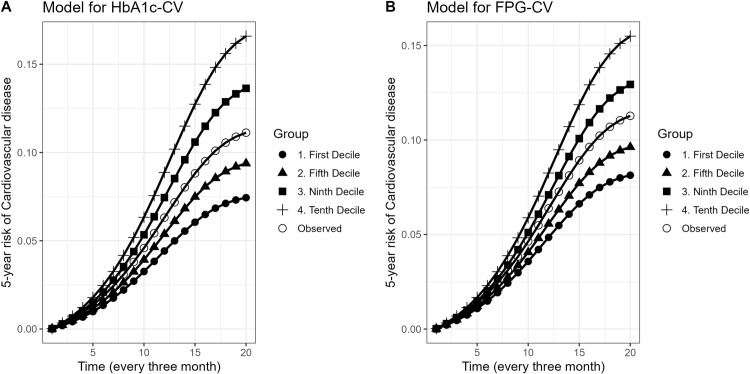
5-year risk of cardiovascular diseases under exposure deciles of A) HbA1C-CV and B) FPG-CV. To avoid plot complexity, we only reported the risk for the first, fifth, ninth and tenth deciles.

In Table 3, we present the 5-year risk, risk ratio and risk difference of CVD across different deciles of exposure to HbA1c-SD, FPG-SD, and HbA1c values. The 5-year risk of CVD for HbA1c-SD increased from 8.01% (95% CI: 7.5, 10.1) in the first decile to 15.2% (95% CI: 14.1, 17.7) in the tenth decile. Similarly, the estimated 5-year risk of CVD related to FPG-SD rose from 8.5% (95% CI: 7.7, 10.3) in the first decile to 14.8% (95% CI: 13, 17.2) in the tenth decile. The 5-year risk of CVD for HbA1c-CV and FPG-CV presented in Fig 4.

**Table 3:** Adjusted 5-year risk of cardiovascular diseases (CVD) under different levels of HbA1c-SD, FPG-SD, and HbA1c value, using parametric g-formula.

Hypothetical intervention	5-year risk of CVD^a^ (95% CI)	Population risk ratio^b^ (95% CI)	Population risk difference (95% CI)	Cumulative percentage intervened on^c^	Average percentage intervened on^d^
**HbA1c-SD**					
**Natural course**	11.03 (10.2, 12.6)	1.37 (1.21, 1.48)	3.02 (2.06, 3.9)	0	0
**Decile 1**	8.01 (7.5, 10.1)	1	0	100	69.18
**Decile 2**	8.4 (7.8, 10.3)	1.04 (1.02, 1.05)	0.39 (0.23, 0.42)	100	93.51
**Decile 3**	8.7 (8.1, 10.6)	1.08 (1.04, 1.11)	0.69 (0.46, 0.84)	100	92.83
**Decile 4**	9.1 (8.5, 10.9)	1.14 (1.07, 1.17)	1.1 (0.75, 1.35)	100	91.08
**Decile 5**	9.5 (8.9, 11.2)	1.19 (1.10, 1.23)	1.5 (1.03, 1.9)	100	91.65
**Decile 6**	10.04 (9.4, 11.6)	1.25 (1.14, 1.31)	2.03 (1.4, 2.5)	100	88.31
**Decile 7**	10.7 (10.01, 12.2)	1.34 (1.18, 1.42)	2.6 (1.84, 3.38)	100	86.19
**Decile 8**	11.6 (10.8, 13.04)	1.45 (1.24, 1.56)	3.6 (2.41, 4.52)	100	81.86
**Decile 9**	12.9 (12.01, 14.7)	1.61 (1.32, 1.77)	4.9 (3.24, 6.26)	100	73.29
**Decile 10**	15.2 (14.1, 17.7)	1.90 (1.46, 2.15)	7.2 (4.63, 9.32)	100	73.46
**FPG-SD**					
**Natural course**	11.1 (10.3, 12.6)	1.30 (1.14, 1.41)	2.6 (1.5, 3.5)	0	0
**Decile 1**	8.5 (7.7, 10.3)	1	0	100	66.93
**Decile 2**	8.8 (8.1, 10.5)	1.03 (1.01, 1.04)	0.3 (0.16, 0.40)	100	93.65
**Decile 3**	9.1 (8.3, 10.8)	1.07 (1.03, 1.08)	0.6 (0.32, 0.73)	100	93.74
**Decile 4**	9.4 (8.7, 11)	1.10 (1.05, 1.13)	0.9 (0.51, 1.1)	100	92.50
**Decile 5**	9.7 (9.03, 11.3)	1.14 (1.07, 1.19)	1.2 (0.71, 1.6)	100	92.14
**Decile 6**	10.1 (9.5, 11.6)	1.19 (1.09, 1.25)	1.6 (0.94, 2.15)	100	90
**Decile 7**	10.7 (9.9, 12.1)	1.25 (1.12, 1.34)	2.2 (1.2, 2.9)	100	86.57
**Decile 8**	11.4 (10.7, 13)	1.34 (1.17, 1.47)	2.9 (1.17, 3.9)	100	81.14
**Decile 9**	12.6 (11.6, 14.4)	1.48 (1.23, 1.67)	4.1 (2.31, 5.7)	100	70.98
**Decile 10**	14.8 (13.02, 17.2)	1.73 (1.34, 2.03)	6.3 (3.4, 8.7)	100	73.21
**HBA1C value in each visit**					
**Natural course** ^*^	10.8 (10.1, 12.2)	2.17 (1.65, 2.40)	5.8 (4.3, 6.7)	0	0
**Low A1C <5**	4.9 (4.5, 6.9)	1	0	100	88.42
**Medium A1C (5 to ≤7)**	7.9 (7.5, 9.6)	1.61 (1.35, 1.71)	3 (2.3, 3.5)	100	56.30
**High A1C (>7) in each**	11.6 (10.8, 13.1)	2.34 (1.73, 2.61)	6.6 (4.5, 7.7)	90.94	25.08

* As a reference (g-form risk under no hypothetical interventions).

^a.^There were 280 cases of CVD among 2078 patients in the cohort. The observed risk (non-parametric estimate) was 11.6%.

^b.^In addition to hypothetical interventions in the model, estimated using parametric g-formula with time-varying covariates: BMI, systolic and diastolic blood pressure, HbA1c, FBS and Total cholesterol, high-density lipoprotein, low-density lipoprotein and Triglyceride, SGL2, other oral medications, GLP1, insulin, antihypertensive drugs, lipid-lowering drugs and anti-platelet drugs; and time-fixed covariate: age, sex, duration of disease, the baseline and lagged value of time-varying covariates.

^c.^Percent of the population need to intervene in at least one of the time periods (visits).

^d.^Average percent of the population need to intervene in a given time period (across all 3-month time visits).

The estimated 5-year risk of CVD for exposure to the low level of HbA1c (<5%) was 4.9% (95% CI: 4.5, 6.9), and the risk of CVD in the high level of HbA1c (> 7%) was 11.6% (95% CI: 10.8, 13.1).

### 3.2. Effect of exposure to GV

As shown in Table 3, the highest adjusted 5-year risk ratio related to tenth decile was 1.90 (95% CI: 1.46, 2.15) for HbA1c-SD and 1.73 (95% CI: 1.34, 2.03) for FPG-SD, compared to the risk of exposure to the first decile. Thus, considering the natural course as a reference, the adjusted 5-year risk ratio for the tenth decile of HbA1c-SD and FPG-SD was 1.38 (95% CI: 1.21, 1.46) and 1.33 (95% CI: 1.17, 1.45) respectively (S1 Table).

### 3.3. Effect of joint intervention

Table 4 shows the results of a joint hypothetical intervention that exposed participants to various deciles of HbA1c-SD and HbA1c value categories simultaneously. The adjusted 5-year risk and risk ratio for exposure in each HbA1c value group rose as the HbA1c-SD deciles increased. For example, the 5-year risk of CVD for joint exposure to a high (> 7%) HbA1c level increased from 8.2% (95% CI: 7.4, 10.5%) in the first decile to 16.2% (95% CI: 14.6, 18.6%) in the tenth decile of HbA1c-SD. The highest adjusted 5-year risk ratio of CVD was 3.51 (95% CI: 2.50, 4.06) for HbA1c >7% and the tenth decile of HbA1c-SD. The joint models with deciles of HbA1c-CV and quartiles of HbA1c-CV/SD are presented in S3–S5 Tables.

**Table 4:** Adjusted 5-year risk of cardiovascular diseases (CVD) under different levels of joint hypothetical intervention on deciles of HbA1c-SD in different levels of HbA1c using parametric g-formula.

HbA1c level and different decile of visit-to-visit HbA1c variability (SD)	5-year risk of CVD^a^ (95% CI)	Population risk ratio^b^ (95% CI)	Population risk difference (95% CI)	Cumulative percentage intervened on^c^	Average percentage intervened on^d^
**Natural course**	10.9 (10.2, 12.6)	2.37 (1.83, 2.66)	6.4 (4.9, 7.3)	0	0
**A1C <5 + Decile 1** ^*^	4.6 (4.1, 6.4)	1	0	100	93.39
**A1C <5 + Decile 2**	4.9 (4.3, 6.7)	1.05 (1.02, 1.06)	0.3 (0.15, 0.32)	100	99.11
**A1C <5 + Decile 3**	5.1 (4.5, 6.9)	1.10 (1.05, 1.12)	0.5 (0.28, 0.62)	100	99.05
**A1C <5 + Decile 4**	5.3 (4.8, 7.3)	1.15 (1.08, 1.19)	0.7 (0.45, 0.98)	100	98.91
**A1C <5 + Decile 5**	5.6 (5.04, 7.6)	1.21 (1.12, 1.26)	1 (0.61, 1.4)	100	99.08
**A1C <5 + Decile 6**	5.9 (5.3, 8.1)	1.28 (1.15, 1.35)	1.3 (0.8, 1.8)	100	98.79
**A1C <5 + Decile 7**	6.3 (5.7, 8.6)	1.38 (1.21, 1.47)	1.7 (1.1, 2.4)	100	98.73
**A1C <5 + Decile 8**	6.9 (6.1, 9.3)	1.50 (1.26, 1.62)	2.3 (1.4, 3.2)	100	98.57
**A1C <5 + Decile 9**	7.8 (6.7, 10.4)	1.68 (1.35, 1.86)	3.2 (1.8, 4.4)	100	98.41
**A1C <5 + Decile 10**	9.4 (7.8, 12.6)	2.04 (1.51, 2.33)	4.8 (2.7, 6.8)	100	99.06
**A1C (5 to ≤7) + Decile 1**	6.4 (5.8, 7.8)	1.37 (1.19, 1.48)	1.8 (1.1, 2.5)	100	82.04
**A1C (5 to ≤7) + Decile 2**	6.7 (6.2, 8.1)	1.44 (1.25, 1.54)	2.1 (1.4, 2.7)	100	96.66
**A1C (5 to ≤7) + Decile 3**	6.9 (6.5, 8.4)	1.51 (1.30, 1.61)	2.4 (1.8, 2.9)	100	96.43
**A1C (5 to ≤7) + Decile 4**	7.3 (6.9, 8.8)	1.58 (1.36, 1.69)	2.7 (2.1, 3.1)	100	95.72
**A1C (5 to ≤7) + Decile 5**	7.7 (7.2, 9.2)	1.66 (1.41, 1.78)	3.1 (2.5, 3.5)	100	96.19
**A1C (5 to ≤7) + Decile 6**	8.1 (7.6, 9.6)	1.76 (1.48, 1.89)	3.5 (2.9, 3.9)	100	94.81
**A1C (5 to ≤7) + Decile 7**	8.8 (8.2, 10.3)	1.88 (1.56, 2.04)	4.2 (3.4, 4.7)	100	94.15
**A1C (5 to ≤7) + Decile 8**	9.5 (8.9, 11.2)	2.05 (1.67, 2.24)	4.9 (4, 5.6)	100	92.72
**A1C (5 to ≤7) + Decile 9**	10.6 (9.8, 12.6)	2.29 (1.83, 2.53)	6 (4.8, 7.1)	100	90.64
**A1C (5 to ≤7) + Decile 10**	12.8 (11.3, 15.4)	2.76 (2.06, 3.09)	8.2 (6.1, 9.9)	100	92.93
**HbA1c level and different decile of visit-to-visit HbA1c variability (SD)**	**5-year risk of CVD**^a^ **(95% CI)**	**Population risk ratio**^b^ **(95% CI)**	**Population risk difference (95% CI)**	**Cumulative percentage intervened on** ^c^	**Average percentage intervened on** ^d^
**A1C (>7) + Decile 1**	8.2 (7.4, 10.5)	1.77 (1.37, 2.01)	3.6 (2.2, 5.3)	100	80.71
**A1C (>7) + Decile 2**	8.5 (7.8, 10.8)	1.84 (1.43, 2.09)	3.9 (2.6, 5.5)	100	95.43
**A1C (>7) + Decile 3**	8.9 (8.2, 11.1)	1.92 (1.50, 2.17)	4.3 (2.9, 5.8)	100	94.90
**A1C (>7) + Decile 4**	9.4 (8.6, 11.4)	2.02 (1.57, 2.27)	4.8 (3.3, 6.1)	100	93.64
**A1C (>7) + Decile 5**	9.8 (9.1, 11.7)	2.12 (1.64, 2.38)	5.2 (3.8, 6.4)	100	93.90
**A1C (>7) + Decile 6**	10.4 (9.7, 12.1)	2.25 (1.72, 2.52)	5.8 (4.3, 6.9)	100	91.26
**A1C (>7) + Decile 7**	11.1 (10.4, 12.6)	2.41 (1.83, 2.71)	6.5 (5, 7.5)	100	89.43
**A1C (>7) + Decile 8**	12.1 (11.3, 13.6)	2.62 (1.97, 3)	7.5 (5.8, 8.5)	100	85.78
**A1C (>7) + Decile 9**	13.6 (12.5, 15.3)	2.94 (2.16, 3.35)	9 (6.9, 10.3)	100	77.92
**A1C (>7) + Decile 10**	16.2 (14.6, 18.6)	3.51 (2.50, 4.06)	11.6 (8.8, 13.5)	100	76.52

* As a reference (g-form risk under no hypothetical interventions).

^a.^There were 280 cases of CVD among 2078 patients in the cohort. The observed risk (non-parametric estimate) was 11.6%.

^b.^In addition to hypothetical interventions in the model, estimated using parametric g-formula with time-varying covariates: BMI, systolic and diastolic blood pressure, HbA1c, FBS and Total cholesterol, high-density lipoprotein, low-density lipoprotein and Triglyceride, SGL2, other oral medications, GLP1, insulin, antihypertensive drugs, lipid-lowering drugs and anti-platelet drugs; and time-fixed covariate: age, sex, duration of disease, the baseline and lagged value of time-varying covariates.

^c.^Percent of the population need to intervene in at least one of the time periods (visits).

^d.^Average percent of the population need to intervene in a given time period (across all 3-month time visits).

### 3.4. Sensitivity analyses

The difference between the observed risk (11.6%) of CVD and the simulated risk (11.03%) under the natural course (no-intervention) was 0.57%, which indicated the satisfactory model specification. In the first scenario, we employed arbitrary ordering and replicated the analysis using other random ordering of variables. Our findings showed that the estimated risk under no intervention changed from 10.6% to 11.03% in the second scenario. However, the estimated risk ratio and risk difference remained robust. The estimated observed 5-year risk under no intervention for HbA1c-SD model, when entering the continuous variables as linear (in the first analysis), quadratic and cubic polynomials as alternate model specifications (in the second), were 11.03% (95% CI: 10.2, 12.6), 10.6% (95% CI:9.9, 12.1) and, 9.8% (95% CI:8.5, 12.2) respectively. the 5-year risk ratio and risk difference did not change considerably; therefore, we did not report the results.

## 4. Discussion

This study used the parametric g-formula to examine the association between long-term glycemic variability, expressed as HbA1c-SD/CV and FPG-SD/CV, and 5-year risk of CVD adjusted for time-varying confounders affected by prior exposure.

Our results indicated that increasing exposure to HbA1c-SD/CV and FPG-SD/CV from the first to the tenth decile increased adjusted 5-year risk and risk ratio of CVD. For example, the 5-year risk of CVD, when the GV variability calculated by HbA1c-SD increased from 8.01% in the first decile to 15.2% in the tenth decile. Based on our predetermined hypothetical intervention, if the HbA1c-SD variability of all patients were in the tenth decile, the 5-year cardiovascular diseases risk would escalate by 7% relative to the first decile. Furthermore, the 5-year risk ratio of CVD for HbA1c-SD increased from 1.04 in the second decile to 1.90 in the tenth decile compared to the first one. However, to simplify the comparison with other studies, we considered the effect of glycemic variability categorized into quartiles of HbA1c-SD/CV, FPG-SD/CV (S2 Table). Our results showed that the estimated adjusted 5-year risk ratio of CVD was 1.23 (95% CI: 1.11, 1.34) for the fourth quartile, with the first quartile of HbA1c-SD serving as a reference. This was marginally less than the hazard ratio of 1.59 reported in a large population-based retrospective cohort study in China [9]. In a meta-analysis of 23 observational studies, HbA1c-SD or CV were significantly associated with macro-vascular complications, with the pooled hazard ratio of 1.36 (1.27, 1.45) for transient ischemic attack, coronary heart disease, and myocardial infarction related to the HbA1-SD [6]. Moreover, in our study the 5-year risk ratio for CVD related to the fourth quartile of FPG-SD, after adjustment for mean FPG was 1.17 (95%: 1.05, 1.28) compared to the first quartile. However, in a population-based prospective cohort study from Iran [20], the hazard ratio of exposure to the fourth quartile of FPG-SD was 1.03 (95% CI: 0.55, 1.93), which indicates there was no significant association among different quartiles of FPG-SD and CVD in diabetes patients. This difference is due to the fact that we have longitudinal repeated measure values of FPG with three-month intervals, which may lead to more accurate estimates of variability, while the aforementioned research only had four measurements of FPG with three-year intervals. Never the less, similar to our findings, in a population-based study in China, the hazard ratio of stroke for exposure to the fourth quartile of FPG-CV was 1.17 (95% CI: 1.03, 1.32) [21]. Thus, in a study by Zhou et al in 2018, their results indicated that FPG-CV was significantly associated with CVD even after adjusting for other risk factors, including mean FPG [22]; however, when we used deciles to classify FPG-SD, the adjusted 5-year risk ratio increased from 1.03 in second decile to 1.73 in the tenth decile.

The reason for this difference is that, when we use deciles for GV instead of quartiles, we have more uniform SD or CV and more uniform risk in each decile. Therefore, when we compare the risks of two extreme groups, for example, comparing the tenth decile to the first one, we have a larger risk ratio than when using the quartiles. Hence, studies that used quartiles to classify GV may underestimate the risk, in terms of the variety of GV in each quartile. Conversely, the absolute and relative risk reductions may not be comparable across studies with varying risk factor distributions, as the parametric g-formula employs the prevalence of each risk factor value in a specific population under study as a weight to estimate the standardized weighted risk [19,23].

As we indicated, glycemic variability could be an independent risk factor for CVD; however, the underlying mechanism of this relationship remains unclear. Variations in blood glucose levels between high and low have been shown to trigger oxidative stress, which can lead to irreversible changes in epigenetics, dysfunction of endothelial cells, chronic inflammation, and insulin resistance. These factors can cause vascular damage and increase the risk of micro- and macro-vascular diabetes-related complications [24–26]. Furthermore, patients with poor quality of life, and poor adherence to treatment [27], tend to have higher glycemic variability.

Therefore, our results show that the estimated 5-year risk and risk ratio of CVD increase with increasing joint exposure to HbA1c-SD and HbA1c value. For example, the highest 5-year risk of CVD was 3.51 (95% CI: 2.50, 4.06), when the participants were jointly exposed to the tenth decile of HbA1c-SD and HbA1c >7%. Similarly, in a study by Pei J conducted on the ACCORD data, the results indicate that, in patients with high glycemic variability and higher value of HbA1c, the risk was increased [8].

In general, various observational studies have found a significant positive association between exposures to HbA1c and FPG variability (measured by SD or CV) and risk of CVD using Cox proportional hazard models [2,7,9]. However, because these observational studies used conventional methods, they require additional assumptions to be interpreted as the effect of hypothetical interventions, and thus, these estimates cannot be directly compared with the results of our study.

Nevertheless, recent studies have shown that, in addition to increasing the risk of CVD and mortality, glycemic variability also increases the risk of hypoglycemia in patients with type 2 diabetes [28–30]. Higher HbA1c variability has been found to increase the risk of myocardial infarction and stroke, even after controlling for severe hypoglycemic events [22]. Nevertheless, some studies that used more aggressive glucose-lowering treatments to reduce the glucose variability also increased the risk of episodes of severe hypoglycemia, which is already known to be a major determinant of CVD events [31–33]. One of the limitations of these studies is that they did not consider unaware and less severe hypoglycemic episodes that may confound the possible association between GV and CVD.

### 4.1. Strengths and limitations

Our study has several strengths: 1) to our knowledge this the first time that the parametric g-formula has been used to estimate the effect of long-term glycemic variability on the risk of CVD, using a population-based longitudinal cohort study with repeated measurements. 2) we also, used deciles in addition quartiles to have more uniform risk in each group. 3) one of the model misspecifications is the “g-null paradox”, which is unavoidable in parametric g-formula [34], to avoid this bias, we used exposure to risk factors that are already known to have an effect.

Thus, we had several limitations: 1) data on several potential confounders, such as diet, physical activity, smoking status, educational level, and episodes of hypoglycemic events, were not recorded in EHR; therefor this may have led to biased estimates due to unmeasured confounding. 2) we could not model competing events in the algorithm of parametric g-formula, because data about death due to CVD as a competing event were not recorded in HER. 3) data about outcomes were recorded based on the patient’s self-report; therefore, the estimated effects may be prone to information bias. 4) we did not perform subgroup analysis on different types of outcomes (stroke, MI, and CHD), and different types of glucose-lowering medications due to small number of CVD events.

### 4.2. Conclusion

In summary, we found that, in patients with type 2 diabetes, after adjusting for time-varying covariates using the parametric g-formula, lowering HbA1c and/or FPG variability as independent risk factors might reduce the risk of CVD. Moreover, we found that even with a stable HbA1c value, the risk of CVD increased by increasing GV.

## Supporting information

S1 Table Adjusted 5-year risk of cardiovascular diseases (CVD) under different levels of exposure to deciles of HbA1C-SD, FPG-SD, and HbA1C value compared to the natural course, using parametric g-formula. (DOCX)

S2 Table Adjusted 5-year risk of cardiovascular diseases (CVD) under different levels of exposure to quartiles of HbA1C-SD, FPG-SD, and HbA1C value compared to the first quartile, using parametric g-formula. (DOCX)

S3. Table Adjusted 5-year risk of cardiovascular diseases (CVD) under different levels of joint hypothetical intervention on deciles of HbA1c-CV in different levels of HbA1c using parametric g-formula. (DOCX)

S4 Table Adjusted 5-year risk of cardiovascular diseases (CVD) under different levels of joint hypothetical intervention on quartiles of HbA1C-SD in different levels of HbA1C value compared to first quartile and HbA1C <5%, using parametric g-formula. (DOCX)

S5. Table Adjusted 5-year risk of cardiovascular diseases (CVD) under different levels of joint hypothetical intervention on quartiles of HbA1C-CV in different levels of HbA1C value compared to the natural course, using parametric g-formula. (DOCX)
