# Supplementary material for: Long-term glycemic variability and the risk of cardiovascular diseases in type 2 diabetic patients: Effect of hypothetical interventions using parametric g-formula in a population-based historical cohort study
Source: PLoS One. 2025 May 28;20(5):e0319975. doi: 10.1371/journal.pone.0319975 (PMC12118876; doi:10.1371/journal.pone.0319975)
Supplement: S1 Table — (DOCX) [file pone.0319975.s001.docx]

**S1 Table.** **Adjusted 5-year risk of cardiovascular diseases (CVD) under different levels of exposure to deciles of HbA1C-SD, FPG-SD, and HbA1C value compared to the natural course, using parametric g-formula**

| **Hypothetical intervention** | **5-year risk of CVD^a^ (95% CI)** | **Population risk ratio^b^ (95% CI)** | **Population risk difference (95% CI)** | **Cumulative percentage intervened on^c^** | **Average percentage intervened on^d^** |
| --- | --- | --- | --- | --- | --- |
| **HbA1C-SD** | | | | | |
| **Natural course** | 11.03 (10.2, 12.6) | 1 | 0 | 0 | 0 |
| **Decile 1** | 8.01 (7.5, 10.1) | 0.73 (0.68, 0.83) | -3.02 (-3.9, -2.06) | 100 | 69.18 |
| **Decile 2** | 8.3 (7.8, 10.3) | 0.76 (0.71, 0.85) | -2.7 (-3.5, -1.8) | 100 | 93.51 |
| **Decile 3** | 8.7 (8.1, 10.6) | 0.79 (0.75, 0.87) | -2.3 (-3.03, -1.6) | 100 | 92.83 |
| **Decile 4** | 9.1 (8.5, 10.9) | 0.83 (0.79, 0.89) | -1.9 (-2.5, -1.3) | 100 | 91.08 |
| **Decile 5** | 9.5 (8.9, 11.2) | 0.86 (0.83, 0.91) | -1.5 (-2, 1.01) | 100 | 91.65 |
| **Decile 6** | 10.04 (9.4, 11.6) | 0.91 (0.89, 0.94) | -0.99 (-1.3, -0.66) | 100 | 88.31 |
| **Decile 7** | 10.7 (10.01, 12.2) | 0.97 (0.96, 0.99) | -0.33 (-0.51, -0.15) | 100 | 86.19 |
| **Decile 8** | 11.6 (10.8, 13.04) | 1.05 (1.03, 1.07) | 0.56 (0.33, 0.73) | 100 | 81.86 |
| **Decile 9** | 12.9 (12.01, 14.7) | 1.17 (1.09, 1.20) | 1.18 (1.1, 2.4) | 100 | 73.29 |
| **Decile 10** | 15.2 (14.1, 17.7) | 1.38 (1.21, 1.46) | 4.2 (5.6, 5.5) | 100 | 73.46 |
| **FPG-SD** | | | | | |
| **Natural course** | 11.1 (10.3, 12.6) | 1 | 0 | 0 | 0 |
| **Decile 1** | 8.5 (7.7, 10.3) | 0.77 (0.70, 0.87) | -2.5 (-3.5, -1.5) | 100 | 66.93 |
| **Decile 2** | 8.8 (8, 10.5) | 0.79 (0.74, 0.89) | -2.3 (-3.1, -1.3) | 100 | 93.65 |
| **Decile 3** | 9.1 (8.3, 10.8) | 0.82 (0.77, 0.90) | -2 (-2.8, -1.1) | 100 | 93.74 |
| **Decile 4** | 9.4 (8.7, 11) | 0.85 (0.80, 0.92) | -1.7 (-2.3, -0.94) | 100 | 92.50 |
| **Decile 5** | 9.7 (9, 11.3) | 0.88 (0.84, 0.93) | -1.3 (-1.9, -0.74) | 100 | 92.14 |
| **Decile 6** | 10.1 (9.5, 11.6) | 0.91 (0.88, 0.95) | -0.9 (-1.4, -0.51) | 100 | 90 |
| **Decile 7** | 10.7 (9.9, 12.1) | 0.96 (0.94, 0.98) | -0.4 (-0.65, -0.22) | 100 | 86.57 |
| **Decile 8** | 11.4 (10.7, 13) | 1.03 (1.01, 1.05) | 0.3 (0.2, 0.6) | 100 | 81.14 |
| **Decile 9** | 12.6 (11.6, 14.4) | 1.14 (1.07, 1.2) | 1.5 (0.8, 2.2) | 100 | 70.98 |
| **Decile 10** | 14.8 (13, 17.2) | 1.33 (1.17, 1.45) | 3.7 (1.9, 5.2) | 100 | 73.21 |

**S1 Table.** **(Continued).**

| **Hypothetical intervention** | **5-year risk of CVD^a^ (95% CI)** | **Population risk ratio^b^ (95% CI)** | **Population risk difference (95% CI)** | **Cumulative percentage intervened on^c^** | **Average percentage intervened on^d^** |
| --- | --- | --- | --- | --- | --- |
| **HBA1C value in each visit** |  |  |  |  |  |
| **Natural course^*^** | 10.8 (10.1, 12.2) | 2.17 (1.65, 2.40) | 5.8 (4.3, 6.7) | 0 | 0 |
| **Low A1C <5** | 5 (4.5, 7) | 1 | 0 | 100 | 88.42 |
| **Medium A1C (5 to ≤7)** | 8 (7.5, 9.6) | 1.61 (1.35, 1.71) | 3 (2.3, 3.5) | 100 | 56.30 |
| **High A1C (≥7) in each** | 11.6 (10.8, 13.1) | 2.34 (1.73, 2.61) | 6.6 (4.5, 7.7) | 90.94 | 25.08 |

*. As a reference (g-form risk under no hypothetical interventions).

^a^. There were 280 cases of CVD among 2078 patients in the cohort. The observed risk (non-parametric estimate) was 11.6%.

^b^. In addition to hypothetical interventions in the model, estimated using parametric g-formula with time-varying covariates: BMI, systolic and diastolic blood pressure, HbA1c, FBS and Total cholesterol, high-density lipoprotein, low-density lipoprotein and Triglyceride, SGL2, other oral medications, GLP1, insulin, antihypertensive drugs, lipid-lowering drugs and anti-platelet drugs; and time-fixed covariate: age, sex, duration of disease, the baseline and lagged value of time-varying covariates.

^c^. Percent of the population need to intervene in at least one of the time periods (visits).

^d^. Average percent of the population need to intervene in a given time period (across all 3-month time visits).
