# Supplementary material for: Long-term glycemic variability and the risk of cardiovascular diseases in type 2 diabetic patients: Effect of hypothetical interventions using parametric g-formula in a population-based historical cohort study
Source: PLoS One. 2025 May 28;20(5):e0319975. doi: 10.1371/journal.pone.0319975 (PMC12118876; doi:10.1371/journal.pone.0319975)
Supplement: S2 Table — (DOCX) [file pone.0319975.s002.docx]

**S2 Table.** **Adjusted 5-year risk of cardiovascular diseases (CVD) under different levels of exposure to quartiles of HbA1C-SD, FPG-SD, and HbA1C value compared to the first quartile, using parametric g-formula**

| **Hypothetical intervention** | **5-year risk of CVD^a^ (95% CI)** | **Population risk ratio^b^ (95% CI)** | **Population risk difference (95% CI)** | **Cumulative percentage intervened on^c^** | **Average percentage intervened on^d^** |
| --- | --- | --- | --- | --- | --- |
| **HBA1C value in each visit** |  |  |  |  |  |
| **Natural course** | 10.8 (10.1, 12.2) | 2.17 (1.64, 2.39) | 5.9 (4.3, 6.7) | 0 | 0 |
| **Low A1C <5^*^** | 4.9 (4.4, 6.7) | 1 | 0 | 100 | 88.42 |
| **Medium A1C (5 to ≤7)** | 7.9 (7.5, 9.6) | 1.61 (1.35, 1.71) | 3 (2.3, 3.5) | 99.99 | 56.31 |
| **High A1C (≥7) in each** | 11.6 (10.8, 13.1) | 2.34 (1.73, 2.61) | 6.7 (4.9, 7.7) | 90.94 | 25.08 |
| **Visit to visit HbA1C variability (SD)** |  |  |  |  |  |
| **Natural course** | 10.8 (10, 12) | 1.17 (1.08, 1.24) | 1.6 (0.9, 2.3) | 0 | 0 |
| **Quartile 1^*^** | 9.2 (8.6, 11) | 1 | 0 | 100 | 71.83 |
| **Quartile 2** | 9.7 (9, 11.4) | 1.05 (1.02, 1.06) | 0.5 (0.3, 0.6) | 100 | 87.66 |
| **Quartile 3** | 10.3 (9.6, 11.9) | 1.12 (1.06, 1.17) | 1.1 (0.6, 1.6) | 100 | 76.59 |
| **Quartile 4** | 11.3 (10.5, 12.9) | 1.23 (1.11, 1.4) | 2.1 (1.2, 3.1) | 100 | 59.11 |
| **Visit to visit FBS variability (SD)** |  |  |  |  |  |
| **Natural course** | 10.8 (10.1, 12.4) | 1.129 (1.04, 1.20) | 1.13 (1.04, 1.20) | 0 | 0 |
| **Quartile 1^*^** | 9.6 (8.7, 11.3) | 1 | 1 | 100 | 71.17 |
| **Quartile 2** | 9.8 (9.1, 11.5) | 1.03 (1.01, 1.05) | 1.03 (1.01, 1.05) | 100 | 90.63 |
| **Quartile 3** | 10.4 (9.6, 11.9) | 1.08 (1.03, 1.13) | 1.08 (1.03, 1.13) | 100 | 79.96 |
| **Quartile 4** | 11.2 (10.4, 12.8) | 1.17 (1.05, 1.28) | 1.17 (1.05, 1.28) | 100 | 57.03 |

**S3 Table.** **(Continued).**

| **Hypothetical intervention** | **5-year risk of CVD^a^ (95% CI)** | **Population risk ratio^b^ (95% CI)** | **Population risk difference (95% CI)** | **Cumulative percentage intervened on^c^** | **Average percentage intervened on^d^** |
| --- | --- | --- | --- | --- | --- |
| **Visit to visit HbA1C variability (CV)** |  |  |  |  |  |
| **Natural course** | 10.8 (10, 12.4) | 1.20 (1.11, 1.29) | 1.8 (1.1, 2.6) | 0 | 0 |
| **Quartile 1^*^** | 9.03 (8.5, 10.9) | 1 | 0 | 100 | 71.91 |
| **Quartile 2** | 9.5 (8.9, 11.3) | 1.05 (1.03, 1.08) | 0.47 (0.34, 0.69) | 100 | 87.61 |
| **Quartile 3** | 10.3 (9.6, 11.8) | 1.14 (1.08, 1.21) | 1.2 (0.89, 1.8) | 100 | 76.67 |
| **Quartile 4** | 11.6 (10.7, 13.1) | 1.28 (1.15, 1.41) | 2.5 (1.6, 3.7) | 100 | 58.13 |
| **Visit to visit FBS variability (CV)** |  |  |  |  |  |
| **Natural course** | 10.9 (10.1, 12.5) | 1.16 (1.07, 1.21) | 1.5 (0.7, 2) | 0 | 0 |
| **Quartile 1^*^** | **9.4 (8.7, 11.1)** | 1 | 0 | 100 | 69.23 |
| **Quartile 2** | 9.8 (9.1, 11.4) | 1.04 (1.02, 1.05) | 0.4 (0.1, 0.5) | 100 | 89.07 |
| **Quartile 3** | 10.4 (9.7, 11.9) | 1.11 (1.04, 1.13) | 1 (0.5, 1.3) | 100 | 81.16 |
| **Quartile 4** | 11.5 (10.6, 13.1) | 1.22 (1.09, 1.29) | 2.1 (0.9, 2.8) | 100 | 56.25 |

*. As a reference (g-form risk under no hypothetical interventions).

^a^. There were 280 cases of CVD among 2078 patients in the cohort. The observed risk (non-parametric estimate) was 11.6%.

^b^. In addition to hypothetical interventions in the model, estimated using parametric g-formula with time-varying covariates: BMI, systolic and diastolic blood pressure, HbA1c, FBS and Total cholesterol, high-density lipoprotein, low-density lipoprotein and Triglyceride, SGL2, other oral medications, GLP1, insulin, antihypertensive drugs, lipid-lowering drugs and anti-platelet drugs; and time-fixed covariate: age, sex, duration of disease, the baseline and lagged value of time-varying covariates.

^c^. Percent of the population need to intervene in at least one of the time periods (visits).

^d^. Average percent of the population need to intervene in a given time period (across all 3-month time visits).
