# Supplementary material for: Long-term glycemic variability and the risk of cardiovascular diseases in type 2 diabetic patients: Effect of hypothetical interventions using parametric g-formula in a population-based historical cohort study
Source: PLoS One. 2025 May 28;20(5):e0319975. doi: 10.1371/journal.pone.0319975 (PMC12118876; doi:10.1371/journal.pone.0319975)
Supplement: S3. Table — (DOCX) [file pone.0319975.s003.docx]

**Table S3.** Adjusted 5-year risk of cardiovascular diseases (CVD) under different levels of joint hypothetical intervention on deciles of HbA1c-CV in different levels of HbA1c using parametric g-formula

| **HbA1c level and different decile of visit-to-visit HbA1c variability (CV)** | **5-year risk of CVD^a^ (95% CI)** | **Population risk ratio^b^ (95% CI)** | **Population risk difference (95% CI)** | **Cumulative percentage intervened on^c^** | **Average percentage intervened on^d^** |
| --- | --- | --- | --- | --- | --- |
| **Natural course** | 10.9 (10.1, 12.6) | 2.9 (2.1, 3.3) | 7.2 (5.7, 8.3) | 0 | 0 |
| **A1C <5 + Decile 1^*^** | **3.7 (3.3, 5.3)** | **1** | **0** | **100** | **96.01** |
| **A1C <5 + Decile 2** | 3.9 (3.5, 5.6) | 1.05 (1.03, 1.06) | 0.2 (0.1, 0.3) | 100 | 99.34 |
| **A1C <5 + Decile 3** | 4.1 (3.7, 5.8) | 1.10 (1.1, 1.07) | 0.4 (0.3, 0.6) | 100 | 99.30 |
| **A1C <5 + Decile 4** | 4.3 (3.8, 6.1) | 1.16 (1.10, 1.19) | 0.6 (0.5, 0.9) | 100 | 99.27 |
| **A1C <5 + Decile 5** | 4.6 (4.1, 6.4) | 1.21 (1.14, 1.27) | 0.9 (0.6, 0.12) | 100 | 99.18 |
| **A1C <5 + Decile 6** | 4.8 (4.3, 6.7) | 1.28 (1.17, 1.35) | 1.1 (8.3, 1.6) | 100 | 99.08 |
| **A1C <5 + Decile 7** | 5.1 (4.6, 7.1) | 1.36 (1.23, 1.47) | 1.4 (1.1, 2.1) | 100 | 98.93 |
| **A1C <5 + Decile 8** | 5.5 (4.9, 7.7) | 1.48 (1.29, 1.61) | 1.8 (1.4, 2.7) | 100 | 98.76 |
| **A1C <5 + Decile 9** | 6.2 (5.7, 8.7) | 1.64 (1.29, 1.84) | 2.5 (1.8, 3.6) | 100 | 98.61 |
| **A1C <5 + Decile 10** | 7.3 (6.5, 10.4) | 1.95 (1.54, 2.27) | 3.6 (2.6, 5.4) | 100 | 99.04 |
| **A1C (5 to ≤7) + Decile 1** | 6.1 (5.6, 7.5) | 1.61 (1.37, 1.73) | 2.4 (1.8, 2.8) | 100 | 86.15 |
| **A1C (5 to ≤7) + Decile 2** | 6.4 (5.9, 7.7) | 1.69 (1.43, 1.81) | 2.7 (2.2, 3.1) | 100 | 97.81 |
| **A1C (5 to ≤7) + Decile 3** | 6.7 (6.2, 8) | 1.77 (1.49, 1.90) | 3 (2.5, 3.4) | 100 | 97.59 |
| **A1C (5 to ≤7) + Decile 4** | 6.9 (6.5, 8.3) | 1.85 (1.55, 1.99) | 3.2 (2.8, 3.7) | 100 | 97.47 |
| **A1C (5 to ≤7) + Decile 5** | 7.3 (6.8, 8.7) | 1.95 (1.61, 2.11) | 3.6 (3.05, 4.1) | 100 | 97.05 |
| **A1C (5 to ≤7) + Decile 6** | 7.7 (7.2, 9.3) | 2.05 (1.67, 2.24) | 4 (3.4, 4.6) | 100 | 96.74 |
| **A1C (5 to ≤7) + Decile 7** | 8.2 (7.6, 9.9) | 2.18 (1.75, 2.42) | 4.5 (3.8, 5.2) | 100 | 96.01 |
| **A1C (5 to ≤7) + Decile 8** | 8.9 (8.2, 10.8) | 2.36 (1.84, 2.65) | 5.2 (4.3, 6.03) | 100 | 95.11 |
| **A1C (5 to ≤7) + Decile 9** | 9.8 (9.1, 11.9) | 2.61 (1.97, 2.99) | 6.1 (5.04, 7.3) | 100 | 93.97 |
| **A1C (5 to ≤7) + Decile 10** | 11.6 (10.6, 14.2) | 3.07 (2.20, 3.63) | 7.9 (6.3, 9.6) | 100 | 95.25 |

**Table S8.** (Continued).

| **HbA1c level and different decile of visit-to-visit HbA1c variability (SD)** | **5-year risk of CVD^a^ (95% CI)** | **Population risk ratio^b^ (95% CI)** | **Population risk difference (95% CI)** | **Cumulative percentage intervened on^c^** | **Average percentage intervened on^d^** |
| --- | --- | --- | --- | --- | --- |
| **A1C (>7) + Decile 1** | 8.8 (8.3, 10.7) | 2.35 (1.77, 2.66) | 5.1 (3.8, 6.3) | 100 | 78.40 |
| **A1C (>7) + Decile 2** | 9.3 (8.7, 11.05) | 2.47 (1.86, 2.78) | 5.6 (4.2, 6.7) | 100 | 96.64 |
| **A1C (>7) + Decile 3** | 9.7 (9.1, 11.4) | 2.58 (1.93, 2.90) | 6 (4.6, 7.05) | 100 | 96.18 |
| **A1C (>7) + Decile 4** | 10.1 (9.5, 11.8) | 2.70 (2.01, 3.03) | 6.4 (5.1, 7.4) | 100 | 95.74 |
| **A1C (>7) + Decile 5** | 10.6 (9.9, 12.3) | 2.83 (2.09, 3.18) | 6.9 (5.5, 7.9) | 100 | 95.33 |
| **A1C (>7) + Decile 6** | 11.2 (10.4, 12.9) | 2.98 (2.18, 3.35) | 7.5 (5.9, 8.5) | 100 | 94.43 |
| **A1C (>7) + Decile 7** | 11.9 (11.01, 13.6) | 3.16 (2.29, 3.56) | 8.2 (6.4, 9.3) | 100 | 93.19 |
| **A1C (>7) + Decile 8** | 12.8 (11.8, 14.6) | 3.41 (2.41, 3.87) | 9.1 (7.1, 10.3) | 100 | 91.32 |
| **A1C (>7) + Decile 9** | 14.1 (12.9, 16.2) | 3.75 (2.57, 4.34) | 10.4 (8.01, 11.9) | 100 | 88.31 |
| **A1C (>7) + Decile 10** | 16.4 (14.6, 18.9) | 4.35 (2.85, 5.17) | 12.7 (9.6, 14.8) | 100 | 89.37 |

*. As a reference (g-form risk under no hypothetical interventions).

^a^. There were 280 cases of CVD among 2078 patients in the cohort. The observed risk (non-parametric estimate) was 11.6%.

^b^. In addition to hypothetical interventions in the model, estimated using parametric g-formula with time-varying covariates: BMI, systolic and diastolic blood pressure, HbA1c, FBS and Total cholesterol, high-density lipoprotein, low-density lipoprotein and Triglyceride, SGL2, other oral medications, GLP1, insulin, antihypertensive drugs, lipid-lowering drugs and anti-platelet drugs; and time-fixed covariate: age, sex, duration of disease, the baseline and lagged value of time-varying covariates.

^c^. Percent of the population need to intervene in at least one of the time periods (visits).

^d^. Average percent of the population need to intervene in a given time period (across all 3-month time visits).
