# Supplementary material for: Long-term glycemic variability and the risk of cardiovascular diseases in type 2 diabetic patients: Effect of hypothetical interventions using parametric g-formula in a population-based historical cohort study
Source: PLoS One. 2025 May 28;20(5):e0319975. doi: 10.1371/journal.pone.0319975 (PMC12118876; doi:10.1371/journal.pone.0319975)
Supplement: S4 Table — (DOCX) [file pone.0319975.s004.docx]

**S4 Table.** **Adjusted 5-year risk of cardiovascular diseases (CVD) under different levels of joint hypothetical intervention on quartiles of HbA1C-SD in different levels of HbA1C value compared to first quartile and HbA1C <5%, using parametric g-formula**

| **HbA1C level and different quartile of visit-to-visit HbA1C variability (SD)** | **5-year risk of CVD^a^ (95% CI)** | **Population risk ratio^b^ (95% CI)** | **Population risk difference (95% CI)** | **Cumulative percentage intervened on^c^** | **Average percentage intervened on^d^** |
| --- | --- | --- | --- | --- | --- |
| **Natural course** | 10.9 (10.1, 12.6) | 2.41 (1.87, 2.66) | 6.4 (5.1, 7.3) | 0 | 0 |
| **Quartile 1 + A1C <5^*^** | **4.5 (4, 6.2)** | 1 | 0 | 100 | 94.61 |
| **Quartile 2 + A1C <5** | 4.8 (4.3, 6.7) | 1.07 (1.05, 1.10) | 0.3 (0.2, 0.5) | 100 | 98.17 |
| **Quartile 3 + A1C <5** | 5.5 (4.9, 7.6) | 1.21 (1.13, 1.28) | 1.1 (0.6, 1.5) | 100 | 97.47 |
| **Quartile 4 + A1C <5** | 6.4 (5.7, 9.1) | 1.43 (1.26, 1.59) | 1.9 (1.3, 3) | 100 | 97.78 |
| **Quartile 1 + A1C (5 to ≤7)** | 6.5 (6.1, 7.8) | 1.44 (1.23, 1.55) | 2 (1.4, 2.6) | 100 | 84.67 |
| **Quartile 2 + A1C (5 to ≤7)** | 7.1 (6.6, 8.4) | 1.57 (1.35, 1.67) | 2.6 (2, 3.1) | 100 | 93.71 |
| **Quartile 3 + A1C (5 to ≤7)** | 8.03 (7.5, 9.6) | 1.78 (1.51, 1.89) | 3.5 (2.9, 4) | 100 | 89.42 |
| **Quartile 4 + A1C (5 to ≤7)** | 9.5 (8.7, 11.6) | 2.10 (1.71, 2.29) | 5 (4.1, 6) | 100 | 86.67 |
| **Quartile 1 + A1C (>7)** | 8.7 (7.9, 10.6) | 1.93 (1.5, 2.2) | 4.2 (2.7, 5.6) | 100 | 82.13 |
| **Quartile 2 + A1C (>7)** | 9.5 (8.7, 11.3) | 2.11 (1.6, 2.4) | 5 (3.6, 6.3) | 100 | 91.33 |
| **Quartile 3 + A1C (>7)** | 10.8 (10.1, 12.4) | 2.39 (1.8, 2.7) | 6.3 (4.8, 6.3) | 100 | 82.33 |
| **Quartile 4 + A1C (>7)** | 12.9 (11.8, 14.9) | 2.86 (2.1, 3.2) | 8.4 (6.6, 9.7) | 100 | 65.47 |

*. As a reference (g-form risk under no hypothetical interventions).

^a^. There were 280 cases of CVD among 2078 patients in the cohort. The observed risk (non-parametric estimate) was 11.6%.

^b^. In addition to hypothetical interventions in the model, estimated using parametric g-formula with time-varying covariates: BMI, systolic and diastolic blood pressure, HbA1c, FBS and Total cholesterol, high-density lipoprotein, low-density lipoprotein and Triglyceride, SGL2, other oral medications, GLP1, insulin, antihypertensive drugs, lipid-lowering drugs and anti-platelet drugs; and time-fixed covariate: age, sex, duration of disease, the baseline and lagged value of time-varying covariates.

^c^. Percent of the population need to intervene in at least one of the time periods (visits).

^d^. Average percent of the population need to intervene in a given time period (across all 3-month time visits).
