# Supplementary material for: Long-term glycemic variability and the risk of cardiovascular diseases in type 2 diabetic patients: Effect of hypothetical interventions using parametric g-formula in a population-based historical cohort study
Source: PLoS One. 2025 May 28;20(5):e0319975. doi: 10.1371/journal.pone.0319975 (PMC12118876; doi:10.1371/journal.pone.0319975)
Supplement: S5. Table — (DOCX) [file pone.0319975.s005.docx]

**S5 Table.** Adjusted 5-year risk of cardiovascular diseases (CVD) under different levels of joint hypothetical intervention on quartiles of HbA1C-CV in different levels of HbA1C value compared to the natural course, using parametric g-formula

| **HbA1C level and different quartile of visit-to-visit HbA1C variability (CV)** | **5-year risk of CVD^a^ (95% CI)** | **Population risk ratio^b^ (95% CI)** | **Population risk difference (95% CI)** | **Cumulative percentage intervened on^c^** | **Average percentage intervened on^d^** |
| --- | --- | --- | --- | --- | --- |
| **Natural course^*^** | **10.9 (10.2, 12.6)** | **1** | **0** | **0** | **0** |
| **Quartile 1 + A1C <5** | 3.70 (3.26, 5.28) | 0.33 (30.03, 46.06) | -7.2 (-8.41, -5.81) | 100 | 96.35 |
| **Quartile 2 + A1C <5** | 4.01 (3.54, 5.66) | 0.36 (32.7, 48.71) | -6.89 (-8.02, -5.51) | 100 | 98.28 |
| **Quartile 3 + A1C <5** | 4.57 (4.06, 6.34) | 0.41 (37.6, 54.59) | -6.33 (-7.32, -4.97) | 100 | 97.04 |
| **Quartile 4 + A1C <5** | 5.49 (4.93, 7.69) | 0.50 (45.6, 65.07) | -5.41 (-6.3, -3.86) | 100 | 96.13 |
| **Quartile 1 + A1C (5 to ≤7)** | 5.96 (5.46, 7.37) | 0.54 (0.50, 0.64) | -4.94 (-5.95, -3.86) | 100 | 87.26 |
| **Quartile 2 + A1C (5 to ≤7)** | 6.51 (6.02, 7.83) | 0.59 (0.56, 0.69) | -4.39 (-5.27, -3.39) | 100 | 94.13 |
| **Quartile 3 + A1C (5 to ≤7)** | 7.47 (6.93, 8.93) | 0.68 (0.65, 0.76) | -3.43 (-4.07, -2.58) | 100 | 89.34 |
| **Quartile 4 + A1C (5 to ≤7)** | 8.99 (8.32, 10.91) | 0.82 (0.79, 0.91) | -1.91 (-2.42, -1.04) | 100 | 83.34 |
| **Quartile 1 + A1C (>7)** | 8.70 (8.09, 10.5) | 0.79 (0.73, 0.88) | -2.2 (-3.01, -1.38) | 100 | 80.12 |
| **Quartile 2 + A1C (>7)** | 9.54 (8.92, 11.26) | 0.87 (0.82, 0.93) | -1.36 (-2.06, -0.77) | 100 | 90.83 |
| **Quartile 3 + A1C (>7)** | 11.0 (10.23, 12.62) | 1 (0.97, 1.03) | 0.1 (-0.32, 0.40) | 100 | 82.31 |
| **Quartile 4 + A1C (>7)** | 13.33 (12.19, 15.30) | 1.21 (1.14, 1.25) | 2.43 (1.16, 2.82) | 100 | 67.25 |

*. As a reference (g-form risk under no hypothetical interventions).

^a^. There were 280 cases of CVD among 2078 patients in the cohort. The observed risk (non-parametric estimate) was 11.6%.

^b^. In addition to hypothetical interventions in the model, estimated using parametric g-formula with time-varying covariates: BMI, systolic and diastolic blood pressure, HbA1c, FBS and Total cholesterol, high-density lipoprotein, low-density lipoprotein and Triglyceride, SGL2, other oral medications, GLP1, insulin, antihypertensive drugs, lipid-lowering drugs and anti-platelet drugs; and time-fixed covariate: age, sex, duration of disease, the baseline and lagged value of time-varying covariates.

^c^. Percent of the population need to intervene in at least one of the time periods (visits).

^d^. Average percent of the population need to intervene in a given time period (across all 3-month time visits).
